# Neuromechanobiology: Bridging Mechanobiology and Neuroscience Through Evidence and Open Questions

**DOI:** 10.3390/cells15020178

**Published:** 2026-01-19

**Authors:** Karolina Zimkowska, Marc Riu-Villanueva, José A. del Río

**Affiliations:** 1 Molecular and Cellular Neurobiotechnology, Institute for Bioengineering of Catalonia (IBEC), Barcelona Institute of Science and Technology (BIST), Science Park of Barcelona, 08028 Barcelona, Spain; kzimkowska@ibecbarcelona.eu (K.Z.); mriu@ibecbarcelona.eu (M.R.-V.); 2 Department of Cell Biology, Physiology and Immunology, Faculty of Biology, University of Barcelona, 08028 Barcelona, Spain; 3 Network Center for Biomedical Research of Neurodegenerative Diseases (CIBERNED), Institute Carlos III, Ministry of Health, 08028 Barcelona, Spain; 4 Institute of Neuroscience, University of Barcelona, 08035 Barcelona, Spain

**Keywords:** Neuromechanobiology, mechanical forces, mechanotransduction, brain development, neural migration and regional specification, axon guidance, synaptic activity

## Abstract

Neuromechanobiology has emerged as a multidisciplinary field at the interface of neuroscience and mechanobiology, aiming to elucidate how mechanical forces influence the development, organization, and function of the nervous system. This review offers a comprehensive overview of the historical evolution of the discipline, its molecular and biophysical foundations, and the experimental strategies employed to investigate it. Recent advances have revealed the pivotal roles of substrate stiffness, mechanical signaling, and force transduction in neural stem proliferation, axon guidance, synapse formation, and neural circuit maturation. All these effects originate at the molecular level and extend to the mesoscopic scale. Disrupted mechanotransduction has been increasingly implicated in neurodevelopmental disorders and neurodegenerative diseases, underscoring its clinical relevance. Key unresolved questions and future directions are also highlighted, with emphasis on the need for integrative approaches to decipher the complex interplay between mechanical forces and neural function.

## 1. Introduction

Mechanobiology is an interdisciplinary field that explores how mechanical cues influence biological processes such as cell proliferation, differentiation, migration, and function [1,2,3,4]. In the nervous system, mechanical signals are essential throughout development, from the earliest stages of neural tube formation to the ongoing regulation of neuronal function and plasticity, aging, and repair [5,6,7,8,9,10]. During embryonic development, mechanical forces sculpt the structural organization of the brain [10,11,12]. In fact, biophysical factors (e.g., hydrostatic pressure), gradients of extracellular matrix (ECM) stiffness, and cell-generated tension drive neural tube closure, cortical folding, and axonal pathfinding [13,14,15,16,17]. For example, when considering differential brain stiffness and neurite migration, some opposing reports described that neurons cultured on stiffer surfaces tend to develop shorter or longer axons ([15,18] see also [19]). However, the forces generated by the growth cone regulate axon development and fasciculation [20]. Indeed, neural progenitor cells are especially sensitive to extracellular cell mechanics, which influence lineage commitment and the spatial arrangement of neural circuits [21,22,23,24,25]. The ability of neurons and glial cells to sense and respond to their mechanical environment is mediated by a range of mechanotransduction pathways, including integrins, cytoskeletal linkers, and mechanically activated ion channels [22,26,27,28,29,30,31,32]. In mature nervous tissue, mechanobiological feedback continues to play a central role. Synaptic activity, dendritic spine morphology, and glial modulation are all affected by mechanical stimuli [33,34,35,36,37].

On the other hand, mechanical signals can regulate neurotransmission and synaptic activity through cytoskeletal remodeling and changes in ion channel conductance [38]. For example, it has been described that stiff substrates increase neuronal activity [39,40,41]. In parallel, astrocytes and microglia, which maintain homeostasis and respond to injury, also rely on mechanosensing to regulate their functions [42,43,44,45,46,47]. Indeed, glial cells play an active and essential role in mechanotransduction within the central nervous system, acting as key sensors and responders to mechanical cues under both physiological and pathological conditions (e.g., [2,5,32,45,48,49]). Astrocytes, microglia, and oligodendrocyte lineage cells express a broad range of mechanosensitive components, including integrins, cytoskeletal regulators, mechanosensitive ion channels, and downstream transcriptional effectors, which enable them to translate changes in tissue stiffness, strain, and extracellular matrix composition into intracellular signaling responses [43,50]. Importantly, numerous studies have demonstrated that glial mechanotransduction becomes particularly relevant following CNS injury, where profound alterations in local mechanical properties—most notably increased stiffness associated with glial scar formation—strongly influence cellular behavior and regenerative outcomes [51,52]. In this context, reactive astrocytes display stiffness-dependent changes in morphology, calcium signaling, inflammatory gene expression, and cytokine release, mechanisms that directly contribute to the inhibition of axonal regeneration after injury. Seminal work has further demonstrated that the mechanical properties of the glial scar, rather than its biochemical composition alone, constitute a major barrier to axon growth, underscoring the mechanobiological basis of regeneration failure in the injured CNS (e.g., [48,51]). Collectively, these findings establish glial cells as key regulators of CNS mechanotransduction and highlight their pivotal role in shaping pathological and repair responses following neural injury.

Disruptions in tissue mechanics are increasingly recognized as contributing factors in neurodegenerative diseases. In conditions such as Alzheimer’s disease (AD) or multiple sclerosis (MS), changes in tissue stiffness and ECM composition can impair cellular communication and mechanosensitive signaling [51,53,54,55]. These alterations may exacerbate inflammation, synaptic loss, and neuronal death. In fact, aging itself is associated with shifts in brain viscoelasticity, potentially predisposing neurons to mechanical stress and degeneration [56,57].

Mechanobiology and mechanosensing are also deeply involved in the progression of brain tumors, particularly glioblastoma multiforme (GBM) [58,59,60,61,62]. GBM cells exploit mechanical signals in their microenvironment to enhance invasion, proliferation, and resistance to therapy (e.g., [59]). Tumor progression is accompanied by ECM stiffening, which activates signaling pathways such as Rho GTPases, focal adhesion kinase (FAK), and Yes-associated protein and transcriptional coactivator with PDZ-binding motif (YAP/TAZ) [61,63,64,65]. These pathways regulate cytoskeletal tension, nuclear deformation, and gene expression, promoting aggressive behavior and stem-like properties in tumor cells [1,61]. Mechanosensitive ion channels like Piezo1 are upregulated in GBM and contribute to calcium influx and downstream oncogenic signaling [66]. The tumor’s ability to remodel its microenvironment through both biochemical and mechanical means enables it to infiltrate healthy brain tissue and evade treatment. Indeed, advanced models using stiffness-tunable hydrogels and organotypic cultures have been developed to study GBM mechanobiology and test potential therapies in physiologically relevant conditions [67,68,69,70].

Altogether, mechanobiology provides a powerful framework for understanding the development, function, and dysfunction of the nervous system. It opens new avenues for diagnostics, regenerative strategies, and targeted treatments that consider not only biochemical cues but also the mechanical forces shaping brain health and disease. In this review, we aim to provide a comprehensive overview of the emerging field of Neuromechanobiology. We briefly examine its historical development, the molecular and biophysical principles underlying the organization of the nervous system, and the most significant findings from recent years, with particular emphasis on the development and maturation of the nervous system.

## 2. Neuromechanobiology: A Brief Historical Perspective

Mechanobiology has become an increasingly important discipline within Neuroscience. Around 1993, the term mechanobiology appeared in the title of a scientific publication [71]. In contrast, the term Neuromechanobiology first appeared in PubMed in a 2021 review [72] and previously in some book chapter (e.g., [73]), although studies on mechanobiology, mechanosensing, and mechanotransduction related to the nervous system, at all scales from subcellular to organ level, have been conducted for several decades. The field’s roots date back over a century, with initial anatomical observations of mechanosensitive structures such as the Golgi tendon organ, identified in the late 19th century, and early theoretical contributions like D’Arcy Thompson’s seminal work On Growth and Form, which posited that physical forces shape biological forms [74]. These early milestones laid a conceptual foundation that bridged physics, biology, and neuroanatomy. By the mid-20th century, experimental investigations characterized sensory neurons’ ability to transduce mechanical stimuli. Studies on muscle spindles [75,76], proprioceptors [77,78], and nociceptors [79,80,81] revealed that neurons respond not only to chemical but also to mechanical cues, establishing the concept of mechanoreception as a fundamental sensory modality [82]. A landmark advancement came when Hudspeth and Corey demonstrated that mechanical deflection of hair cell bundles in the inner ear caused rapid ionic currents through mechanically gated ion channels, providing direct evidence of mechanical transduction at the molecular level (see [83,84] for reviews).

Next decades saw rapid progress in identifying molecular components of mechanosensation, including mechanically activated potassium channels such as TREK and TRAAK [85,86,87], alongside advances in understanding the cytoskeleton’s role in force transmission through models like the tensegrity framework (e.g., [88]). These studies highlighted how mechanical forces integrate with biochemical signaling pathways to regulate neural cell architecture and function. Developmental research further revealed that mechanosensitivity in sensory neurons emerges in temporally distinct waves, with mechanoreceptors and proprioceptors acquiring mature mechanotransduction earlier than nociceptors, which depend on target-derived growth factors for maturation [89,90,91]. This underscores the complexity and dynamic nature of mechanobiological processes in nervous system development. In the 21st century, technological innovations such as atomic force microscopy, traction force microscopy, and microfabricated substrates have enabled precise measurement and manipulation of mechanical forces at cellular and subcellular scales (e.g., [7,92,93]). These tools have expanded mechanobiology’s relevance, elucidating its roles in neuronal plasticity, injury response, and pathologies, including neurodegenerative diseases and brain tumors. Understanding mechanobiological mechanisms promises new therapeutic strategies targeting mechanical signaling pathways in the nervous system (please see [54,94,95] for putative therapeutic strategies). However, such strategies should be carefully considered in light of their potential off-target or collateral effects, as mechanotransduction-related receptors, ligands, and signaling pathways are broadly expressed and functionally active across multiple tissues and organs, rather than being restricted to the nervous system (see [96] or [97] for additional comments). Thus, Neuromechanobiology stands as a vital interdisciplinary frontier that bridges biomechanics, molecular neuroscience, and clinical neurology, providing fresh perspectives on how physical forces shape neural health and disease [73].

## 3. Decoding the Molecular Symphony of Mechanotransduction in the Nervous System: From Mechanical Signals, Ion Channels to Cytoskeletal Dynamics

### 3.1. Mechanics of the Brain

**Figure 1:**
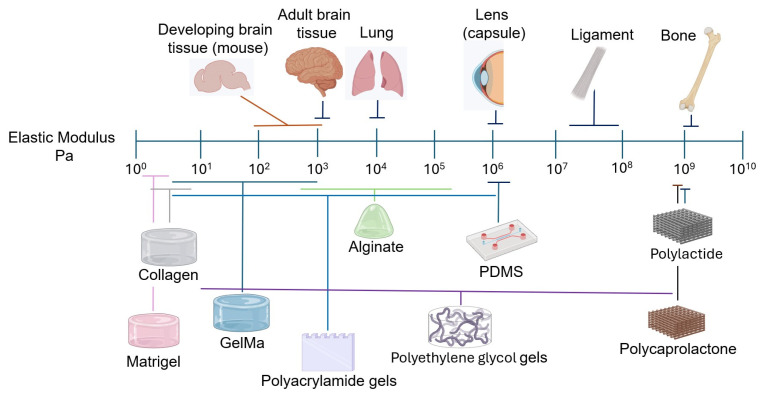
Scheme illustrating the values of the elastic modulus in Pa for some tissues as well as for various currently used biomaterials. The values shown were obtained from the following studies. Developing brain [117,118] other tissues [98,119], PDMS [120], GelMa [121], Alginate [122], Matrigel^TM^ [118], other biomaterials [123].

The brain is an exceptionally soft and complex tissue, often regarded as the softest in the human body [98] (Figure 1). Protected from most external mechanical stimuli by the skull, its mechanical properties rely on the large proportion of proteoglycan-bound water and lipids, and on the relatively low content of fibrous proteins (e.g., collagen and fibronectin) compared to other tissues [25,72,98,99]. Although some data indicate no relevant changes in the stiffness of the mouse brain during postnatal development (e.g., [100]) or a decrease in stiffness with aging [101], it is generally assumed that, although brain stiffness is different between mice and higher mammals, including humans, stiffness increases with age, starting from development [102,103,104,105,106]. For example, during development, brain cortical stiffness increases from approximately 0.1–1 kPa in early gestation to around 0.5–2 kPa at birth, while adult brain cortex stiffness typically ranges between 2 and 5 kPa, depending on the region and measurement method, with white matter being stiffer than grey matter [107,108,109,110]. Regarding its mechanical anisotropy, the brain has been described as isotropic, anisotropic, or as exhibiting a mixture of both properties, depending on the study (see [104] for details). At very small strains, brain tissue behaves as a linear viscoelastic material, with both elastic and viscous moduli increasing with frequency in rheological measurements [111]. Beyond this range, the response becomes nonlinear, with stiffness depending on strain and loading type, exhibiting strain-stiffening behavior. Small deformations produce a soft elastic response, whereas larger elastic deformations lead to a pronounced increase in stiffness, characteristic of nonlinear elastic networks [104,112,113]. At high strain levels, this increased stiffness may coincide with thresholds for tissue damage, contributing to its susceptibility to injury [114,115,116].

### 3.2. Mechanosensing in Brain Cells

**Figure 2:**
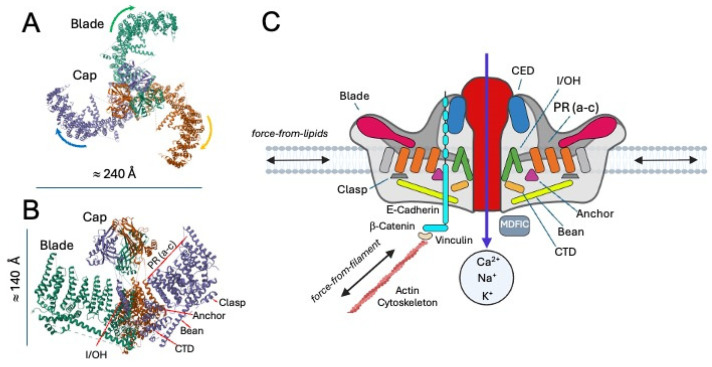
(**A**,**B**) The structure of Piezo1 is from PDB https://doi.org/10.2210/pdb6LQI/pdb structure ID: 6LQI (**upper** view) (**A**) and https://doi.org/10.2210/pdb6BPZ/pdb structure ID: 6BPZ (**side** view) (**B**). The most relevant domains of Piezo1 are indicated. (**C**) Schematic representation showing the arrangement of the main domains of Piezo1 in an open conformation, as well as the described interactions with the actin cytoskeleton via E-cadherin and the interaction with MDFIC, as described in several studies. As indicated in the text, the opening of Piezo channels depends not only on forces derived from changes in plasma membrane tension; Piezo channels can also modulate their conformational states in response to mechanical forces originating from the intracellular cytoskeleton, which are modulated by accessory proteins. Abbreviations: CED, C-terminal extracellular domain; CTD, intracellular C-terminal domain; I/OH, inner and outer helices; PR (a–c), Piezo transmembrane repeats A–C. The illustration was created using PDB data and designed in Biorender (available here: https://BioRender.com/ki8ia0h) modified from [150,151,152].

Neurons and glial cells are continuously exposed to a variety of mechanical signals. They actively sense and respond to mechanical cues in their environment, such as stiffness, viscosity, and spatial constraints. At the same time, they experience forces arising from the activity of neighboring cells, fluid dynamics (e.g., Cerebrospinal fluid (CSF)), or larger-scale tissue deformations (e.g., [36,52,118,124,125,126,127]). Key players in neural mechanosensing are mechanosensitive ion channels (MSICs), which act as primary molecular transducers. Mechanosensitive channels identified in the nervous system comprise epithelial sodium channels (ENaC)/Degenerins (DEG) (which are not present in mammals), transient receptor potential (TRP) channels, the TWIK-related potassium channels, belonging to the two-pore domain potassium (K2P) family, as well as Piezo channels (Table 1, see [128,129]). The Piezo family, particularly Piezo1 and Piezo2, is a critical component for detecting mechanical forces such as membrane stretch, shear stress, and pressure in neurons and glial cells [43,50,130,131,132,133,134,135]. Cryo-EM studies and liposome reconstitution have successfully determined the high-resolution 3D structures of the Piezo channels (e.g., [136,137]) (Figure 2). Each functional Piezo channel is a trimer, with three identical subunits arranged around a central ion-conducting pore [137,138,139]. Every subunit contains 38 transmembrane helices, organized into a series of curved “blade” domains that extend radially from the pore like the arms of a propeller. This distinctive curvature is not merely a structural curiosity: it is thought to bend the surrounding lipid bilayer, creating a dome-shaped membrane footprint that is exquisitely sensitive to changes in tension. When mechanical forces such as membrane stretch, shear stress, or indentation distort this dome, the conformational rearrangements in the blade domains are transmitted inward toward the central pore, triggering its opening [97,133,140,141]. Once activated, both Piezo channels function as non-selective cation channels, permitting the influx of Na^+^ and Ca^2+^ and, to a lesser extent, K^+^ [133,140] (Figure 2). This model is the membrane curvature-dependent gating model [137,141]. By integrating structural analysis, electrophysiological experiments, and molecular dynamics simulations, researchers have recently proposed four distinct conformational states of Piezo: a curved-closed state, an intermediate state, a putative open state, and a flattened-inactivated state (see [138,142] for details). The resulting depolarization can directly trigger action potentials in excitable cells or activate downstream calcium-dependent signaling cascades in non-excitable tissues (see [143] for a recent review). Piezo1 activation and inactivation are rapid and involve distinct mechanisms that ensure proper channel opening, mediated through interactions with accessory proteins [144,145,146], membrane lipids [147], pore lipids [137], and the cytoskeleton [148]. For example, although described in fibroblasts, a MyoD family inhibitor protein (MDFIC) has recently been identified as an auxiliary subunit of Piezo channels, capable of slowing channel inactivation and reducing mechanosensitivity [144]. Additionally, an interaction between Piezo1 and E-cadherin has been reported [148], highlighting the crucial role of actin cytoskeleton integrity in Piezo1 gating [149]. Importantly, these observations indicate that Piezo1 mechanosensitivity is not solely determined by lipid bilayer tension but is also critically modulated by direct coupling to the actin cytoskeleton via the cadherin-β-catenin complex and accessory proteins. This physical tethering allows the channel to detect and transduce long-range mechanical forces transmitted through cell–cell adhesions, supporting a force-from-filament model [129] that complements the classical force-from-lipids paradigm [129], which is also modulated by MDFIC. Although additional mechanisms are likely to be discovered, such dual or currently undescribed gating processes provide Piezo channels with the versatility to integrate multiple mechanical inputs, enhancing their functional adaptability across diverse cellular contexts [148]. Nevertheless, the existence of similar regulatory mechanisms in neural tissue remains to be determined (Table 2).

As indicated, increasing evidence indicates that Piezo1 is expressed across multiple cellular populations of the nervous system, including dorsal root ganglion neurons [153,154], Schwann cells [155,156], reactive astroglia [50], neural stem and progenitor cells [157,158], oligodendroglia [159,160], and microglia [161,162]. Accumulating evidence links its activity to multiple physiological and pathological contexts, underscoring its relevance in both healthy and pathological nervous systems. In contrast, Piezo2 is highly enriched in somatosensory neurons [163], especially those in dorsal root ganglia (DRG) [164,165], Schwann cells [155] and specialized skin mechanoreceptors such as Merkel cells [166,167,168], where it converts light touch, vibration, and proprioceptive cues into patterned neural activity transmitted to the nervous system [135,169] (See Table 1 for comparison with other mechanosensing ion channels).

**Table 1:** Ionotropic mechanosensors in the nervous system. Comparative summary of main families of mechanosensitive channels, properties, functions and cell types where they are present in the nervous system. In bold are outlined the channel family names.

Channel Family	Mechanical Stimuli They Transduce	Ion Permeability	Neural Cell Types Where Present	Described Physiological Function
**Piezo**; Includes Piezo 1 and Piezo 2	Membrane stretch, shear stress and pressure [43,50,130,131,132,133,134,135].	Mainly Na^+^ and Ca^2+^, also K^+^ [133,140].	Piezo 1: NPCs/NSCs, DRG neurons, reactive astroglia, Schwann cells, oligodendrocytes and microglia. Piezo 2: Somatosensory DRG neurons, Schwann cells, Merkel cells [50,153,154,155,156,157,158,159,160,161,162,163,164,165,166,167,168,169].	Wide range of physiological processes, from Piezo 1 sensing Aß fibrils in microglia [162] to Piezo 2 control of gastrointestinal transit in somatosensory neurons [163].
**K2P**; Includes TREK1, TREK2 and TRAAK	Membrane curvature generated by asymmetric tension [170].	K^+^ [171].	Widespread in neurons but also on glial cells in the CNS. Expressed in the PNS’s sensory neurons [172,173].	Maintenance and regulation of resting membrane potential. Modulation of sensory pathways by reducing neuronal excitability [174].
**TRP**; Subfamilies include TRP A/C/M/MC/N/P/V	Polymodal, from osmotic swelling to substrate stiffness [175].	Ca^2+^, Mg^2+^, Na^+^ and [175].	Present in neurons of the sensory and nociceptive pathways, mostly in PNS sensory neurons [176].	Transducers of sensory pathways of pain and heat, through mechanical and chemical sensing. E.g., TRPM8 is activated by temperature, cooling compounds and pressure [176,177].

Complementary to Piezo channels, mechanically activated ion channels include members of the K2P family. Within this group, potassium channel subfamily K member 2 (TREK1 or KCNK2) was the first mammalian ion channel identified as mechanosensitive [178]. Later, two additional K2P channels with similar properties were characterized: potassium channel subfamily K member 10 (TREK2 or KCNK10) [179] and TWIK-related arachidonic acid-stimulated K^+^ channel or potassium channel subfamily K member 4 (TRAAK or KCNK4) [171]. Activation of K2P channels reduces neuronal excitability in sensory pathways by hyperpolarizing the membrane, and their opening can be triggered by asymmetric tension generated through membrane curvature [170] (see also [174] for review). Another group of channels: The TRP channels form a large family with several subfamilies: TRPA (Ankyrin), TRPC (Canonical), TRPM (Melastatin), TRPML (Mucolipin), TRPN (NO-mechano-potential, NOMP), TRPP (Polycystin), and TRPV (Vanilloid). Members such as TRPA1, TRPC1, TRPV2, and TRPV4 act as polymodal mechanosensors, capable of detecting diverse stimuli ranging from osmotic swelling to substrate stiffness, while often integrating both chemical and mechanical signals. Diverse stimuli activate them and are permeable to a range of cations, including Ca^2+^, Mg^2+^, Na^+^, and K^+^ (e.g., [180,181,182,183,184] see also [175]). Additionally, Transmembrane protein 150C (TMEM150C or Tentonin 3), a member of the large “Transmembrane proteins” (TMEM) family, has been suggested to mediate slowly inactivating mechanosensitive currents in proprioceptive neurons of the DRG in mice [185]. However, heterologous expression of TMEM150C alone does not produce mechanically activated currents in cells lacking Piezo1, indicating that it may not act as a pore-forming channel by itself [186]. Further evidence supports the idea that TMEM150C functions more broadly as a general regulator rather than as a stand-alone mechanosensitive channel [187]. A comprehensive review of the functional properties of TMEM family ion channel members, including their physiological roles in both healthy and diseased nervous systems, can be found here [188]. To this point, all the mechanotransduction mechanisms described in these paragraphs are ionotropic, operating rapidly through the activation of ion channels. Summary of the channels and their described regulators can be found in Table 1 and Table 2.

**Table 2:** Regulators of mechanosensing channels. The main mechanosensory regulators that are described in the text with a summary of their function and relevance.

Mechanosensory Regulator	Described Physiological Function
MDFIC	Regulates activation of Piezo channels in different cell types [144].
E-Cadherin	Mediates interaction of Piezo channels with the actin cytoskeleton [148,149].
TMEM150C	Mediates slowly mechanosensitive currents in proprioceptive DRG neurons, acts as a general regulator [187].
STOML3	Scaffolding protein that modulates the activity of mechanosensitive ion channels (e.g., Piezo 1 and 2) by altering membrane mechanics. Essential for normal touch sensation in mammals [189].

In contrast, a slower mechanism also plays a role—the metabotropic mechanotransduction, which relies on G-protein-coupled receptors (GPCRs) that respond to mechanical stimuli like shear stress [190,191]. Several systems, including the sensory system, exhibit GPCR-mediated mechanotransduction [28]. For example, Latrophilin-2 (LPHN2/ADGRL2), expressed in utricular hair cells, is responsible for equilibrium [192] in parallel to GPR133 [193], GPR68 that is present in DRG neurons [194], as well in vascular tissue [195], or GPR126 that plays a crucial role in peripheral nervous system development, particularly Schwann cell myelination [196,197,198]. Despite increasing evidence of their significance, the role of mechanosensitive GPCRs in the nervous system remains largely unexplored, although relevant functions have been described in GBM progression (e.g., GPR133, ADRGE5, or GPR56/ADGRG1) [199,200,201,202].

### 3.3. Integrating Mechanical Signals in Neural Cells

The cytoskeleton acts as a crucial intracellular conduit for mechanical signals; actin filaments [203], microtubules [204,205] and intermediate filaments [206,207,208] form dynamic networks that transmit extracellular forces from focal adhesion complexes inward, modulating mechanosensitive signaling [209]. Integrins, transmembrane receptors that link ECM components like laminins, fibronectin, and collagen to the actin cytoskeleton, serve as mechanotransducers by activating FAK and Src-family kinases, thereby initiating signaling pathways such as RhoA/ROCK and MAPK cascades. These pathways regulate cytoskeletal remodeling, cell adhesion, and gene transcription, which are vital for processes including neurite outgrowth, synaptic plasticity, or myelination (e.g., [73,210,211,212,213]). Beyond ligand binding, integrins can also be triggered by variations in membrane tension induced by mechanical stress, as well as by actomyosin-dependent cytoskeletal forces conveyed through adaptor proteins [214,215]. In addition, integrin-mediated signaling is also known to depend on inside-out activation [209]. Thus, integrins can regulate adhesion and cytoskeletal dynamics bidirectionally, enabling precise cellular responses to extracellular signals (see [215] for review). In this scenario emerged the motor–clutch model originally developed to describe how cells convert cytoskeletal dynamics into mechanical forces during migration and adhesion [216]. In this model, based on the role of focal adhesion, the “motor” corresponds to myosin II activity [217] that generates retrograde actin flow in the leading edge of the cell, while the “clutch” represents dynamic adhesion molecules (e.g., integrins and associated proteins) that transiently engage the actin cytoskeleton to the extracellular matrix [218]. This interplay determines whether actin polymerization results in forward protrusion, retrograde flow, or traction force generation, thereby providing a mechanistic framework for understanding cell motility and force transduction in different substrates [219]. On stiff substrates, rapid tension build-up causes detachment between adhesions and actin filaments, a phenomenon known as frictional slipping. In contrast, on compliant substrates, slower tension development prolongs the engagement of the clutch with the actin network, thereby increasing the cumulative tension until a load-and-fail event occurs [220]. Consequently, the maximum traction force is achieved when the lifetime of a new clutch engagement matches the overall cycle time of the molecular clutch system, corresponding to the optimal balance between force generation and detachment (see [221] for a review of recent advances in the motor–clutch modeling). This model and its modifications [220] have been instrumental in explaining mechanosensing phenomena such as durotaxis [222], negative durotaxis [223,224,225], curvotaxis [226], growth cone motility [218,227], and substrate-dependent migration [220]. However, the model is not considered by the scientific community to be a universal explanation of cell mechanics. Its classical formulation is often viewed as a simplified approximation, as it does not fully incorporate biochemical signaling pathways, membrane mechanics, or the stochastic variability observed in dynamic adhesions [228]. Moreover, certain cell types, such as rapidly migrating immune cells (e.g., neutrophils), among others, exhibit motility behaviors that deviate from motor–clutch predictions, suggesting the involvement of additional or alternative mechanisms [229]. To address these discrepancies, several extensions have been proposed, including stochastic and feedback-based clutch models, which better account for the diversity of cellular responses across mechanical environments (e.g., viscoelastic substrates) [219]. Moreover, the contribution of the microtubule cytoskeleton is not examined in detail, despite its well-established role in regulating GBM tumor cell motility and other cellular processes across different cell types (see below) [230].

**Figure 3:**
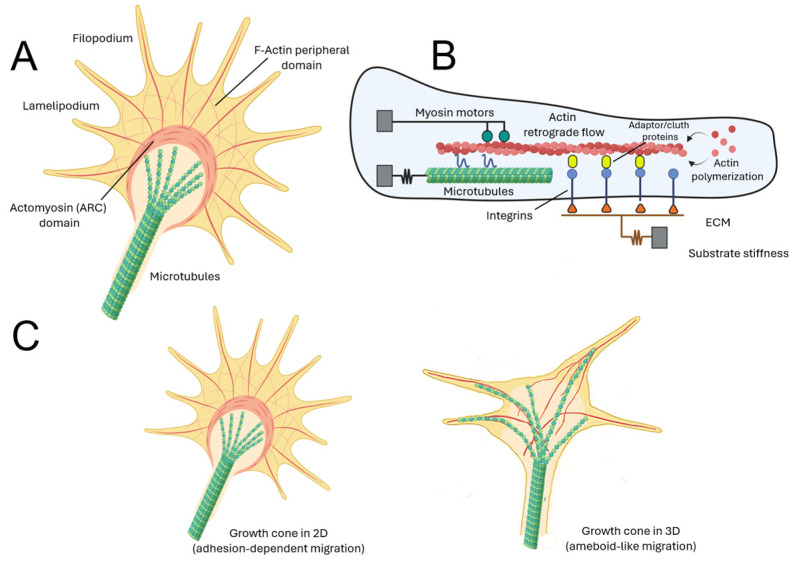
(**A**) The axonal growth cone is classically divided into three main domains: the central domain (not shown), the transitional zone (Actomyosin (ARC) domain), and the peripheral domain. Actin filaments are most abundant in the peripheral domain, where they form dense branched networks in lamellipodia and parallel bundles in filopodia, providing structural support and driving protrusive activity. Myosin II is enriched in the transitional zone, where it generates contractile forces that regulate actin retrograde flow and mediate the coupling between actin filaments and microtubules extending from the central domain. (**B**) In the motor–clutch model, the axonal growth cone’s dynamics are explained by the interplay between actin polymerization, molecular motors, and substrate adhesion. Actin filaments in the peripheral domain polymerize at the leading edge, pushing the membrane forward, while retrograde flow driven by myosin II motors pulls the actin network rearward. Transmembrane adhesion molecules (e.g., integrins), often linked to the extracellular matrix, act as “clutches” with the help of an adaptor protein (e.g., talin or vinculin) that can engage or disengage with the retrograding actin filaments. But also, a dynamic equilibrium is established between integrin–ECM binding. In the classical model, when clutches engage, they transmit traction forces to the substrate, slowing retrograde flow and allowing forward protrusion of the growth cone. When they disengage, retrograde flow accelerates, reducing traction and leading to retraction. The clutch model predicts the existence of an optimal stiffness/ECM density in which cells’ force and migration are maximal. (**C**) In two-dimensional (2D) cultures, neuronal growth cones tend to be large, flattened, and rich in filopodia and lamellipodia, expanding across the substrate and forming distinct peripheral and central domains; for example, sensory neuron growth cones on 2D collagen often exceed 200 µm^2^ in projected area, with abundant protrusive structures. By contrast, in three-dimensional (3D) matrices like collagen gels, growth cones are significantly smaller—often around a third the size—with fewer lamellipodia and filopodia, and exhibit morphologies more akin to those seen in vivo. Moreover, whereas 2D growth cones typically maintain discrete actin- and microtubule-defined domains, in 3D the domain segregation becomes blurred: growth cones lack distinct partitions, displaying an actin-rich front with filopodia but without actin arcs or lamellipodia, and microtubule tips extend much closer to the leading edge. Please note that growth cones adopting an amoeboid mode of motility are relatively smaller than growth cones observed in two-dimensional cultures. In the schematic, both are displayed at a comparable magnification to facilitate visualization and comparison of cytoskeletal organization. The illustration was modified from [221,227,235,241].

In Neuroscience, the motor–clutch model has been especially influential in explaining the biomechanics of the neuronal growth cone [218,231]. Growth cone motility relies on actin-dynamics and substrate adhesions, and the motor–clutch framework has successfully explained how axons sense substrate stiffness, regulate protrusion–retraction cycles, and integrate mechanical with chemical guidance cues [218,227,232]. Beyond neurons, extensions of this model have also been applied to glial cell migration, including astrocytes and progenitor cells during nervous system development or repair [24,233,234]. However, since the work published by F. Bradke’s laboratory, it has become clear that the migratory behavior of axonal growth cones strongly differs between 2D and 3D substrates (see Figure 3) [235]. In 3D environments, hippocampal growth cones extend axons independently of substrate pulling forces without requiring adhesions [209]. In this context, the 3D amoeboid-like mode of migration aligns with the predictions of the motor–clutch model in durotaxis. Under such conditions, more closely resembling the in vivo situation, the cytoskeletal organization and functional roles within the growth cone differ markedly from those observed in 2D. Specifically, both microtubule and actin cytoskeleton dynamics exhibit distinct features in 3D compared to 2D. Although actin retrograde flow and microtubule growth speed were found to be similar in both settings, neurons cultured in 3D hydrogels displayed microtubule ends protruding closer to the leading edge of growth cones, in contrast to the more restricted localization observed in 2D [235]. For these reasons, key microenvironmental features of the growth cone, including the composition and mechanical properties of the extracellular matrix as well as cytoskeletal dynamics, must be carefully considered when interpreting its behavior. These factors should be taken into account in experimental designs that aim to compare data obtained from two-dimensional and three-dimensional systems, where force transmission, confinement, and cytoskeletal organization differ substantially. Along similar lines, a recent study from R. Mayor’s group, employing Walker 256 carcinosarcoma cells in microchannel confinement models, described a migration paradigm independent of focal adhesions, termed frictiotaxis. In this model, durotactic migration relies on an asymmetric distribution of myosin and actomyosin retrograde flow. The authors proposed that the mechanism driving this focal adhesion-independent durotaxis is that stiffer substrates provide greater friction [236]. In fact, increasing levels of confinement favor adhesion-independent migration in many healthy cell types (e.g., dendritic cells, neutrophils, lymphocytes, or Schwann cells [237,238,239,240]. Nevertheless, although the mechanisms described in both studies may exhibit similarities with growth-cone dynamics in 3D contexts, frictiotaxis has not yet been directly demonstrated in neural cells. Thus, systematic experiments in neural cells will be necessary to assess the relevance of friction-dependent mechanisms in both cellular migration and neuritic dynamics.

Thus, these observations point to microtubule dynamics as an additional player in mechanotransduction processes. Most of these studies have focused on models of traumatic brain injury [242], axonal lesion [243,244], or neurodegenerative disease (e.g., [53]). In summary, current knowledge indicates that crucial elements of microtubule dynamics, such as the microtubule-associated protein Tau, play an important role (see, for example, [245] for review). In this context, it has been reported that mechanical force alone can induce Tau mislocalization to dendritic spines and a loss of synaptic function in in vitro neuronal cultures with random cell organization [246,247]. In fact, this mislocalization of Tau leads to postsynaptic deficits, partly due to the internalization of α-amino-3-hydroxy-5-methyl-4-isoxazolepropionic acid (AMPA) receptors in the affected dendritic spines, impairing synaptic function [246,247]. In parallel, the negative effects of pathogenic Tau on the actin cytoskeleton and microtubules cause a toxic destabilization of the lamin nucleoskeleton and formation of nuclear invaginations and blebs [248,249,250]. These structural changes often precede apoptosis, gene dysfunction, or premature cellular aging [251,252]. De facto, anti-Tau strategies, in addition to targeting its aggregation or intracellular transport [253], have recently shifted their focus toward its nuclear effects to correct changes in genetic expression and cell death. In this context, a recent study reported a large-scale screening of small molecules designed to suppress Tau-mediated nuclear pathologies in human frontotemporal dementia (FTD) neurons. From a library of 20,000 small molecules, the screen identified more than 20 compounds that corrected the phenotype in a dose-dependent manner [254]. In this context, and as a link between the cooperative activity of the actomyosin axis and microtubules, several studies have also focused on the molecular connections between these two cytoskeletal components. One of the most extensively studied molecules is Spectrin. A recent study using expansion microscopy revealed that, in fibroblasts, distinct Spectrin meshwork densities coexist inside cells. Through biophysical measurements and computational modeling, it was shown that the non-polarized Spectrin network, with the intervention of actomyosin, can dynamically transition into polarized clusters fenced by actin stress fibers, resembling the periodic arrays observed in neurons [255]. In this regard, Swaim et al. demonstrated that mechanotransduction signaling, regulated by RhoA and Spectrin, controls the distribution of axonal microtubule polymers in response to forces exerted on the axon by muscle contractility [256]. Regarding the third fundamental component of the cytoskeleton, the intermediate filaments, several studies have linked their mechanical properties to their involvement in mechanotransduction processes [207,257]. However, studies specifically addressing their role in Neuromechanobiology remain limited and require more comprehensive and in-depth investigation. Most of the existing research has primarily focused on Vimentin or Keratin expressed in other cell types, rather than on their specific functions within the nervous system.

### 3.4. Hippo Signaling at the Crossroads of Cytoskeletal Dynamics and Neuronal Mechanotransduction

**Figure 4:**
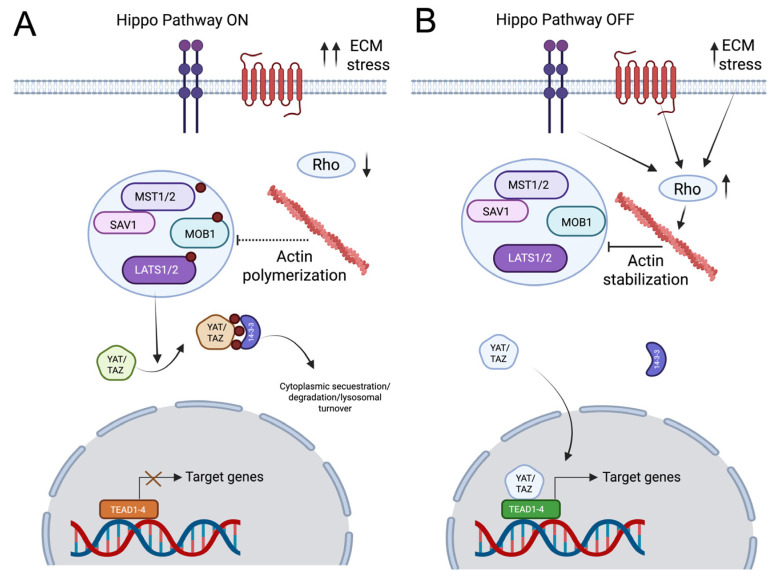
A graphical summary of Hippo signaling. Although several components have been identified across different physiological contexts, in neurons, the extracellular stress, such as mechanical strain or ATP release, is sensed by integrins and purinergic GPCRs (P1 and P2Y receptors), converging on cytoskeletal and stress–response pathways. (**A**) Chronic extracellular stress or excessive purinergic signaling favors activation of MST1/2 through phosphorylation, which in turn phosphorylates and activates LATS1/2. Active, phosphorylated LATS1/2 phosphorylate YAP/TAZ, retaining them in the cytoplasm and, after additional phosphorylation events, lead to polyubiquitination and proteasome-mediated degradation. The phosphatase PP2A counteracts this process by dephosphorylating MST1/2, LATS1/2, or YAP/TAZ depending on the context, serving as a regulatory brake that can buffer stress-induced Hippo activation. (**B**) In conditions of moderate stress, Integrin engagement increases actin tension and increases RhoA balance in its GTP-bound state, which stabilize actomyosin cytoskeleton and indirectly suppresses Hippo kinase activity. Under these conditions, LATS1/2 remain largely unphosphorylated, allowing YAP/TAZ to remain dephosphorylated, translocate into the nucleus, and activate survival and plasticity gene programs. In fact, substrate stiffness modulates the frequency of Rho GTPase activation and generation of protrusions at least in adult neural stem cells [265]. Through this phosphorylation–dephosphorylation balance, integrins, purinergic GPCRs, and PP2A jointly determine whether extracellular stress drives adaptive neuronal remodeling or degenerative outcomes. Created in BioRender (available here: https://BioRender.com/1l91fz6).

The Hippo signaling pathway, from the Drosophila gene hippo, whose loss-of-function mutations caused tissue overgrowth resembling an organ “as big as a hippopotamus” [258], is a highly conserved kinase cascade that orchestrates aspects of neuronal development and physiology by precisely regulating the transcriptional coactivators YAP and TAZ [259]. In neurons, the pathway is initiated by Mammalian Sterile 20-like protein kinase 1/2 (MST1/2) kinases, which, upon autophosphorylation at Thr183/Thr180, associate with the scaffolding protein Salvador homolog 1 (SAV1), facilitating recruitment and phosphorylation of the Large Tumor Suppressor 1/2 (LATS1/2) kinases at Thr1079/Thr1041. Activated LATS1/2 subsequently phosphorylate YAP at Ser61, Ser109, Ser127, Ser164, Ser381, and TAZ at Ser66, Ser89, Ser117, Ser311, generating binding sites for 14-3-3 proteins and promoting cytoplasmic sequestration, ubiquitin-mediated degradation via β-TrCP E3 ligase, or lysosomal turnover. Dephosphorylation processes mediated by intracellular phosphatases such as PP2A and PTPN14 dynamically counteract these phosphorylations, allowing context-dependent nuclear translocation of YAP/TAZ. Mechanical cues sensed through integrin-mediated adhesion, focal adhesion proteins (vinculin, talin, paxillin), and actomyosin contractility regulated by RhoA-ROCK signaling inhibit Hippo kinase activity, thereby stabilizing nuclear YAP/TAZ (at least during embryo development) [260,261] (please see Figure 4 for more details of Hippo signaling). Once in the nucleus, YAP/TAZ associates with TEAD1-4 (in tumor cells [262]) or SMAD2/3 (in fibroblasts [263]), or with the miR-29a promoter to induce expression of genes governing neurite outgrowth (e.g., GAP43 and NF-200) in Neuroblastoma cell lines [264].

In addition to transcriptional regulation, YAP/TAZ directly interfaces with the actin cytoskeleton, binding to F-Actin, Angiomotin (AMOT), and angiomotin-like proteins (e.g., AMOTL1 and AMOTL2), which both sense cytoskeletal tension and feed back to modulate YAP/TAZ localization [266,267,268]. Post-translational modifications such as acetylation of Lys494/Lys497 by the histone acetyltransferases p300 and CBP enhance nuclear retention [269], while SUMOylation and methylation [270] fine-tune transcriptional activity in a context-dependent manner (see [271] for a recent review). Hippo/YAP signaling is integrated with other key pathways, including Wnt/β-catenin [272,273], Notch [274], mTOR [275], MAPK [276], or Sonic hedgehog (SHH) [277]. Although these interactions have been described mainly in developmental processes and tumorigenesis, they are very likely also to occur in the normal and/or pathological nervous system (see below). In fact, during early cortical development, overactivation of YAP has been shown to cause excessive ectopic production of radial glial cells and the formation of heterotopias [278,279] or enlarged neocortex with abnormal folding [280] as also occurs in the developing cerebellum [281]. Furthermore, inactivation of LATS1 and LATS2, the main negative regulators of YAP/TAZ activity, leads neural progenitor cells to transiently over-proliferate, followed by rapid and extensive cell death [282].

## 4. Mechanotransduction in Neurons and Glial Cells

In the following paragraphs, a general overview of the most relevant findings in the field of developmental Neuromechanobiology is provided. In this context, recent data on the roles of mechanosensing and mechanotransduction in governing the physiology of neural progenitors, their dynamic behavior during the process of neural tube formation, and their subsequent functional development in the cerebral cortex as a model system are examined.

### 4.1. Neural Stem Cell Proliferation and Cortical Lamination

Neural stem cells (NSCs) integrate multiple signals to determine cell fate. In fact, mechanical properties such as stiffness, elasticity, and topography of the ECM have been shown to influence NSC proliferation and lineage commitment, with softer matrices generally favoring NPC self-renewal and stiffer environments promoting neuronal and glial differentiation [283,284]. This is a dynamic process that is modulated during development, as demonstrated by Ryu et al. [285]. These extracellular signals are integrated through intracellular mechanotransduction pathways in which YAP and TAZ act as central effectors. YAP/TAZ nuclear localization is enhanced in response to increased matrix stiffness and cell-ECM adhesion, thereby promoting transcriptional programs that sustain proliferation. Conversely, activation of the Hippo pathway leads to YAP phosphorylation and cytoplasmic retention, ultimately limiting NSC expansion [286,287]. Thus, the interplay between ECM biomechanics and Hippo/YAP signaling constitutes a critical regulatory axis in NSC biology. Multiple studies carried out in pluripotent cells from different species, including humans, have shown their roles in maintaining NSCs’ self-renewal. In the developing chick neural tube, overactivation of YAP markedly increased the pool of neural progenitors in a TEAD-dependent manner [288]. Conversely, YAP/TAZ is expressed by neural progenitor cells and is required for maintaining the proliferative potential and structural organization, and its ablation during neocortical development reduces the number of cortical projection neurons in the upper cortical layer and causes the loss of ependymal cells, resulting in hydrocephaly [279,289]. Similar results were determined in the developing cerebellum [281].

**Figure 5:**
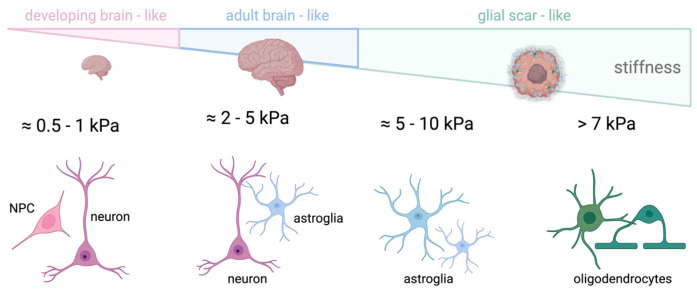
Influence of matrix stiffness on human neural progenitor cell (NPC) fate. Substrate stiffness can govern NPC lineage specification by modulating mechanosensitive signaling. Soft matrices (0.5–1 kPa) that mimic the compliant embryonic developing cortex, such as low-crosslink PEG-diacrylate (~0.7 kPa), dilute (0.5%) hyaluronic acid or Matrigel (~0.3–0.5 kPa), or low-percentage collagen I (~0.4 kPa), maintain NPCs’ proliferative capacity and favor neuronal differentiation. The limited integrin tension in soft matrices keeps RhoA/ROCK activity low and YAP/TAZ cytoplasmic, allowing basal Wnt/β-catenin and TRPC1 activity to promote neurogenesis (β-III-tubulin^+^/MAP2^+^ neurons). Intermediate stiffness (2–5 kPa)—recapitulated by PEG-DA, GelMA, collagen/alginate, or fibrin hydrogels—matches adult brain parenchyma mechanics (≈2–4 kPa) and induces balanced mechanotransduction: partial YAP/TAZ nuclear entry with TRPV4-mediated Ca^2+^ influx activates the JAK/STAT3 astroglial pathway, while TRPC1-Wnt/Akt signaling maintains neuronal output. NPCs thus yield mixed neuronal and glial populations. Furthermore, the exact balance can be modulated by external stimuli, e.g., adding stretch favors glia, while microgrids or electrical stimulation favors neurons. Stiff matrices (>5 kPa) such as high-crosslink PEG-DA, polyacrylamide (5–10 kPa), or dense collagen gels, mimic reactive-glial scar tissue (≈6–12 kPa). Strong focal-adhesion formation drives high RhoA/ROCK activity, nuclear YAP/TAZ, and maximal TRPV4-dependent JAK/STAT3 as well as Shh/Gli1 (oligodendroglial) activation, while TRPC1/Wnt signaling is suppressed, promoting GFAP^+^ astrocytic and OLIG2^+^/MBP^+^ oligodendroglial differentiation. Neuronal markers are reduced, and the cells display a more flattened, stress-fiber-rich morphology. Created with Biorender (available here: https://BioRender.com/koq15cs).

Thus, in an attempt to better understand the role of ECM stiffness in NSC renewal and differentiation, several in vitro approaches have been developed. For instance, Leipzig et al. reported that NSCs cultured on methacrylamide chitosan (MAC) hydrogels displayed maximal proliferation at ~3.5 kPa, with neuronal differentiation favored on very soft substrates (<1 kPa) and oligodendrocytic outcomes enhanced on stiffer matrices (>7 kPa) (Figure 5) [283]. Similar findings were observed in 3D alginate hydrogels, where neuronal marker expression (β-tubulin III (TUJ1)) increased on soft gels resembling brain tissue [290]. More recently, NSCs cultured in hyaluronic acid hydrogels with tunable elasticity (17–250 Pa) showed distinct behaviors depending on stiffness: softer matrices supported long-term growth and neuronal differentiation, whereas stiffer ones promoted early adhesion and later glial commitment [291]. Variable-modulus polymer networks further confirmed that neurogenesis is maximized on substrates of 100–500 Pa, while glial cells dominate at 1000–10,000 Pa (Figure 5) [284]. Beyond stiffness alone, the combination of mechanical cues and topographical features can further guide NSC fate, as demonstrated by PAA-ACA platforms where increased rigidity, together with microgrids, enhanced neuronal differentiation and neurite maturation [292]. Consistently, 3D graphene scaffolds revealed that rigid substrates (~64 kPa) bias NSCs toward astroglial fates and suppress neuronal differentiation compared with softer matrices (~30 kPa) [293].

An important line of experimental research has focused not only on identifying biochemical factors but also on elucidating the biomechanical mechanisms involved in neurulation, or neural tube formation, and neural crest formation using several models. Readers interested in a comprehensive and up-to-date overview of the molecular mechanisms governing neural tube development may consult these reviews [294,295]. The complex dynamics of mechanical forces during mammalian neurulation were described by Galea et al. Using laser ablation at a single point of the closing neural tube, Galea and colleagues demonstrated that this was enough to reopen a section of it longitudinally [296]. Notably, following this reopening, tissues located far from the ablation site exhibited expansions or compressions, indicating a mechanical connection between these distant tissues and the closing neural tube mediated by the actin cytoskeleton [296]. Regarding neural crest induction (also referred to as competence), beyond the well-known biochemical factors such as BMP, Wnt, and FGF, hydrostatic pressure has emerged as an important regulator of neural crest competence. In an elegant study, R. Mayor’s laboratory demonstrated this by manipulating hydrostatic pressure in vivo. They showed that an increase in pressure in the blastocoel inhibits YAP signaling and, consequently, disrupts Wnt activation in the responding tissue—both processes being essential for proper neural crest induction [297]. In fact, these phenotypes are rescued by increased nuclear β-catenin but not by its cytoplasmic accumulation. The study shows that increased hydrostatic pressure during blastocoel expansion leads to higher cell density, resulting in cytoplasmic retention of YAP and a loss of competence. Since the vitelline membrane restricts embryo growth, the rise in pressure forces cell compaction rather than volume expansion [297]. In fact, it was shown that high confluence of mammalian cells leads to the activation of LATS and the phosphorylation and inactivation of YAP [298]. Moreover, and importantly, the intrinsic degree of fluidity between regions of the hindbrain and the spinal cord accounts for the differences in folding and expansion observed at the dorso-caudal level in chicken embryos during development [299]. Finally, one of the aspects that has long been used as a model is the migration of neural crest cells. With this process in mind, it has been determined that, in addition to the chemotaxis described in various studies (see [300] for review), neural crest cells also migrate influenced by the stiffness of the surrounding substrate [301,302]. In this regard, it has been shown that these cells remodel the ECM and its stiffness to find the environment most suitable for their migration [301,302].

### 4.2. Modeling Neural Stem Cell Proliferation and Neural Tube Closure in Brain Organoids

Cerebral organoids, first pioneered by Lancaster and colleagues in 2013 [303], have become transformative tools for modeling early human brain development [304,305,306]. Two major approaches exist: unguided protocols, which rely on the intrinsic self-organization of pluripotent stem cells to generate heterogeneous brain-like regions [307,308], and guided protocols, which use patterning cues such as dual-SMAD inhibition, Wnt, or SHH modulation to direct differentiation toward specific neural lineages [307,309,310]. The use of organoids has increased greatly in recent years. In fact, several studies have generated neural tube-like structures in vitro from mouse and human pluripotent stem cells [311,312,313]. Beyond lineage specification, recent work has highlighted how physical forces, matrix stiffness, and actomyosin contractility shape tissue morphogenesis and neural tube-like patterning in organoids [314]. Together, these insights underscore that brain organoids are not only biochemical but also biophysical systems, where mechanical forces orchestrate morphogenesis, symmetry breaking, dorso-ventral polarization, and ultimately the fidelity of neurodevelopmental modeling in vitro. For example, a study identifies NUAK2 (a serine/threonine protein kinase that belongs to the AMPK-related kinase family) as a critical regulator of human neural tube closure. Researchers reported a consanguineous family with recurrent cases of anencephaly, in which exome sequencing uncovered a homozygous loss-of-function mutation in NUAK2. Using human induced pluripotent stem cell-derived neural progenitors and cerebral unguided organoids, the authors demonstrated that NUAK2 deficiency disrupts Hippo/YAP signaling, leading to aberrant cytoplasmic retention of YAP and defective apical actomyosin contractility. These defects impair the apical constriction and elongation of neuroepithelial cells, processes essential for neurulation. Collectively, the work provides the first genetic and mechanistic evidence linking NUAK2 activity to neural tube morphogenesis in humans, underscoring how alterations in mechanobiological pathways can directly cause severe developmental disorders such as anencephaly [315]. The application of organoids in Neuromechanobiology research has expanded to encompass a wide range of purposes.

## 5. From Mechanical Signals to Brain Architecture (i): Mechanotransduction in Cortical Gyrification and Brain Regionalization

Cortical gyrification, defined as the folding of the cerebral cortex into sulci and gyri, first appears in gyrencephalic species (such as carnivores and large rodents) and becomes progressively more pronounced in primates, reaching its highest complexity in the human brain [316]. Cortical development in rodents (agyrencephalic) and humans (gyrencephalic) brains begins with NSCs in the lower ventricular zone, but the diversity and dynamics of these progenitors differ significantly across species. In rodents, apical radial glia (aRG) are the principal progenitors, dividing at the ventricular surface to generate neurons directly or through intermediate progenitors (IPs) located in the subventricular zone. These IPs typically undergo a limited number of divisions before differentiation, supporting the rapid and relatively simple lamination of the rodent cortex [17]. In humans, while aRGs and IPs are also present, the expansion of the outer subventricular zone introduces an additional population of progenitors, known as outer radial glia (oRG) [317]. These cells are capable of extensive self-renewal and neurogenic divisions, greatly amplifying neuronal output and enabling the dramatic expansion of cortical surface area between gyri and sulci. Thus, this region-specific and prolonged activity of oRGs in humans, compared with their scarcity in rodents, underlie the emergence of cortical gyrification and the evolutionary increase in neuronal diversity and connectivity [318]. Thus, the human neocortex exhibits a folded geometry that enhances network capacity by increasing surface area within the cranial vault. Indeed, gyrification is driven not only by genetic programs controlling progenitor proliferation [318,319,320]. Classic morphoelastic models (e.g., constrained cortical expansion (CCE)) show that modest differences in tangential growth of the cortical plate versus subplate/white matter can trigger buckling instabilities that seed gyri and sulci [320,321,322]. These growth fields are themselves sculpted by progenitor dynamics, ECM composition, axonal tethering, and mechanotransduction. The synthesis below foregrounds those mechanical threads that tie genes to geometry [322,323]. In addition, the classic “tension-based morphogenesis or tension-induced growth (TIG)” hypothesis posits that longitudinal tension along axons/dendrites helps compact wiring and could shape folds [324]. While axons do bear tension, measurements and modern models indicate that axonal tension alone does not set fold topology [321]; rather, it likely acts with, or is downstream of, differential growth to bias patterns and stabilize formed folds. The current consensus is a hybrid view: growth-driven buckling provides the primary instability; axonal and glial process tensions, plus regional material properties, fine-tune placement and maintenance.

### 5.1. Brain Regionalization

Brain regionalization is again a multi-layered process integrating transcriptional programs, signaling gradients, and biomechanical inputs [325,326,327]. While classical models have emphasized morphogens in cortical arealization [325,327,328,329,330], physical properties of neural tissues are equally critical in shaping regional boundaries and guiding neuronal trajectories [10,127,331]. Quantitative measurements of cortical mechanics illustrate striking regional heterogeneity. Magnetic resonance elastography (MRE) has shown that the human occipital cortex exhibits mean stiffness values of ~3.12 ± 0.26 kPa, significantly higher than the lateral occipital cortex (~1.99 ± 0.18 kPa) [110]. Similarly, in high-resolution MRE, the superior parietal cortex demonstrates a mean shear stiffness of ~236 ± 35 Pa, compared to ~192 ± 12 Pa in the postcentral gyrus and ~214 ± 23 Pa in the occipital cortex [332]. Developmental studies further reveal dynamic changes: from childhood to adulthood (ages 5–35), both stiffness and damping ratios evolve differently across cortical regions. Notably, the occipital lobe shows a 30.6% increase in damping ratio, compared to 20.1% in the parietal lobe [333]. This steeper rise suggests that occipital regions undergo greater maturation in their viscoelastic properties, potentially aligning with the establishment of their specialized circuitry. These mechanical distinctions are not trivial. A stiffer occipital cortex may enhance radial glial scaffolding and promote laminar precision needed for visual circuits, whereas parietal regions, characterized by greater compliance, may allow broader dendritic arborization and multisensory integration. The interregional mechanical properties of the cortex may plausibly arise from molecular and biophysical factors that have been progressively integrated into its developmental program over the course of evolution. Nevertheless, the mechanisms underlying the partitioning between the outer telencephalic (pallial) and inner (subpallial) domains remain largely unresolved, underscoring the need for innovative experimental approaches and further in-depth studies.

### 5.2. Pallial–Subpallial Domains: Mechanics and Migration

To the general knowledge and from a biophysical view, the pallium–subpallium boundary illustrates how biomechanics reinforce molecular segregation. In embryonic mouse brains (E12.5–E14.5), atomic force microscopy reveals that the pallium exhibits higher apical stiffness (~1487 Pa at E12.5, decreasing to ~1327 Pa at E14.5), compared to the ganglionic eminence (GE) (~710 Pa at E12.5, dropping to ~541 Pa at E14.5) [117,334]. These differences have been correlated with neural progenitor density (r = 0.99 in the pallium; r = 0.93 in the GE), underscoring a link between cellular packing, stiffness, and domain identity. The stiffer pallium favors radial migration and cortical lamination, while the softer GE facilitates tangential interneuron migration into the pallium, a hallmark of subpallial contribution to cortical inhibitory networks [335,336]. Mechanical inputs at this boundary also interact with ECM dynamics. Laminin-rich basal lamina and actomyosin-generated contractility create anisotropic forces that restrict progenitors to their respective compartments. In fact, stiffness differences in dorsal vs. ventral developing neocortex have also been described during early mouse development (e.g., [117,118,334]). With respect to humans, a study by Hiscox et al. reported an atlas of the viscoelastic properties of the adult human brain, which will undoubtedly be of great relevance for future investigations in both normal and pathological conditions [110]. In addition, data from developmental studies reported that the brainstem was found to be approximately 2–3 times stiffer than both gray and white matter from corona radiata and thalamus (from 5 to 22 months [107]), with similar results from adolescent vs. adult [337]. These differences reflect the progressive changes (from molecules to mesoscopic scales) in the brain associated with evolutionary and adaptive processes in response to various stimuli and functions that have been specifically enhanced across different species [338].

## 6. From Mechanical Signals to Brain Architecture (ii): Mechanosensing During Neuronal Functional Maturation

### Environment, Axonal Growth, and Development of Neuronal Activity

As discussed in the previous sections, the growth cone presents a cytoskeletal structure that is clearly designed to explore the extracellular environment, guided by both chemical and mechanical cues [339]. During axon elongation, different types of neurons responded differently to substrate stiffness. Softer substrates were shown to promote better neurite growth and branching in spinal cord and hippocampal neurons (e.g., [125,340]), but not in cortical [341] or DRG neurons [342]. In this context, and specifically during injury processes, the Piezo1 receptors translocate to the growth cone and mediate the inhibition of its dynamics, thereby preventing axonal regrowth [343]. Therefore, it is reasonable to consider that growth cones act as functional “hubs” for the integration of epigenetic, molecular, and biophysical information [339]. On the other hand, in most studies, the parameters used to model extracellular stiffness or other cues perceived by the growth cone vary considerably. In many cases, these studies present only a single “snapshot” of a dynamic process. However, it has been demonstrated in vivo that changes in cellular proliferation and fluid dynamics can modify and modulate the behavior of growing axons [344], and both the measurement techniques and methodological approaches employed differ across numerous studies (see [93] or [32]). On the other hand, the behavior of the growth cone is different in 2D vs. 3D substrates, as well as its functional activity as described above.

Concerning neural activity linked to membrane depolarization and action potential generation, several studies over the past decades have provided relevant insights. In this regard, one study using induced pluripotent stem cells, rodent neurons, and postmortem human fetal brain neurons illustrated, despite the inherent limitations of cell culture, the remarkable speed with which rodent neurons can develop spontaneous activity. While human-derived cultures exhibited neuronal activity (measured by Ca^2+^ imaging) at day 20 [345], rodent primary cultures showed it already from days 4–7 (measured by Ca^2+^ imaging or multielectrode arrays (MEA) [345,346,347,348]. Additionally, this process leads to much earlier synchronization of the culture in the case of rodent neurons. Given these data and from a mechanobiology point of view and considering factors such as the stiffness or the topography of the substrate where they are cultured, we can make some comments. (i) Biologically, the intrinsic processes of neuronal development and maturation progress more rapidly in rodents than in humans; (ii) It is clearly demonstrated that different neuronal domains showed different rheological properties, the soma behaves like a soft elastic solid, whereas neurites are stiffer and more viscous [349]; (iii) concerning topography, topographically guided cultures displayed more diverse spatiotemporal activity patterns compared to flat substrates. Functional modules emerged that reflected the underlying geometry (e.g., microchannels), and wave-like activity propagation was shaped by the patterned surfaces. On flat surfaces without topography, activity was more globally coherent and highly synchronized (e.g., [350]); (iv) from ECM molecules it is known that substrates enriched with laminin generate a significant increase in activity depending on their stiffness, compared to substrates enriched with fibronectin [351]; and (v) various studies show that neurons growing on compatible but rigid substrates exhibit higher activity due to an increase in voltage-gated calcium channels [40]. In addition to these aspects, we must keep in mind that cell density plays a fundamental role, since higher cell density leads to faster neuronal interconnection and, consequently, quicker activity and synchronization (e.g., [352]). Although substrate stiffness has been reported to play a role in neuronal activity at the level of firing rate, a recent study combining atomic force microscopy (AFM) and MEA measurements demonstrated that no strong correlations between soma and neurite stiffness and mean neuronal firing rate were observed in this preparation [35]. This technically relevant study also showed that although mechanical stiffness of neuronal compartments does not correlate with activity, transient mechanical stimuli on neurons can evoke action potentials and modulate firing rates, indicating that neuronal networks can differentially respond to specific mechanical cues [35], corroborating previous data [36,52]. In this regard, a recent study from the laboratory of R. Luttge demonstrated, using microfabrication and substrate deformation techniques, the activation of neurons in response to deformation processes. These results are in line with previous observations and confirm that topographical modifications of the substrate can modulate neuronal activity [353]. However, a recent study using patch clamp electrophysiology, calcium imaging, and immunofluorescence, reported that hippocampal neurons cultured on stiffer substrates showed a delay in voltage-gated ion channel activity, spontaneous and evoked action potentials, and synapse formation [354].

## 7. Can 3D Cultures and Lab-on-Chip Devices Accurately Model Mechanotransduction in Neuronal Activity?

While 2D factors such as substrate deformability, stiffness, the presence of fluids, and cell density have been analyzed as modulators of neuronal activity (see above), to date, and particularly when considering the differences between 2D and 3D systems, no studies have provided a detailed and dynamic analysis of the changes that occur in the nervous system within 3D contexts as activity patterns evolve. Unfortunately, no established method exists to provide brain organoids with long-term scaffolding that adapts as they grow, as happens in vivo. As organoids expand, scaffolds must dynamically accommodate this growth without limiting development or compromising structural integrity.

In an effort to develop new models, several studies have examined developmental trajectories using organoids derived from distinct brain regions through differential hiPSC differentiation, exploring how maturation unfolds and how these processes can be adapted or modified in model systems (e.g., [355,356,357,358]). Nevertheless, it is important to acknowledge that the current development of brain organoids still faces significant limitations as indicated (e.g., [359,360,361]), including the absence of functional vascularization (e.g., [362]), the low degree of myelination achieved [363], and the intrinsic temporal constraints of functional organoid maturation (e.g., [364,365]). These limitations have been clearly observed in studies aiming to recapitulate pathological conditions of the central nervous system (e.g., [366]). Therefore, data obtained from 2D and 3D systems must be carefully evaluated and interpreted in detail. Nevertheless, Trujillo et al. provided a detailed characterization of the development and evolution of neuronal-derived brain waves in cortical organoids [309]. They found that at around 2 months, organoids exhibited sporadic, isolated spikes with little network organization. By 3–4 months, network bursts appeared, showing synchronized firing across electrodes. Between 6–9 months, complex oscillatory patterns with nested oscillations emerged, resembling delta and theta rhythms observed in preterm human electroencephalogram (EEG) [309]. These waves are typically indicative of high network synchronicity, where neurons fire in a coordinated manner and their presence demonstrates that organoid neuronal networks progressively mature in vitro, modeling aspects of early human cortical development [309]. These observations have also been described from mouse-derived organoids [367]. However, gamma oscillations, reflecting a more complex and mature network state, can only be detected in assembloids of interconnected dorsal and ventral forebrain neurons. The resulting network displays enhanced gamma rhythms and structured bursting activity, not observable in non-connected organoids ([368,369,370] see [371] for a recent review). With respect to whether we can model the effect of mechanical forces on neuronal activity in cortical organoids, a manuscript by Beltrán et al. focused on applying uniaxial compression to human cerebral organoids to mimic traumatic brain injury (TBI) conditions [372]. Using a custom-designed mechanical stimulation system, the researchers applied controlled mechanical forces to the organoids and analyzed their neural responses, demonstrating that as the loading strain rate increased for each sample, the mean Ca^2+^ intensity also increased over time in affected neurons [372]. Unfortunately, no functional analyses were performed in this study to assess the impact of mechanical stimulation on neuronal activity; incorporating approaches such as calcium imaging or electrophysiological recordings would be particularly valuable to address this limitation. Thus, while several studies have demonstrated that mechanical cues can be applied to organoids (e.g., [373]), the impact of such stimuli on network activity, firing patterns, or synaptic function remains largely unexplored, currently being focused on issues of proliferation or differentiation. As indicated, the spatial organization and the properties of the ECM are also crucial for essential developmental and maturational processes. Among other issues, to effectively incorporate exogenous ECM, a comprehensive map of the developing brain’s “matrisome” is required. This knowledge, with advanced biofabrication approaches with ECMs featuring tunable mechanical and biochemical properties, could enable precise control over organoid geometry and the spatial–temporal presentation of extracellular cues, ultimately guiding neural organoid development and functionality.

## 8. Conclusions and Future Directions

Mechanical forces are increasingly recognized as fundamental regulators of brain function and pathology, yet traditional neuronal culture models frequently overlook them as key modulators of cellular and network activity. In this context, there is an urgent need for innovative approaches that, guided by consensus and standardized criteria, can provide the research community with robust tools to explore these phenomena. Such strategies are especially compelling, as mechanical cues have the power to shape neuronal development and functionality under both physiological and pathological conditions, revealing mechanisms that remain hidden in conventional experimental setups. The challenges are significant, yet technological progress is advancing even faster. However, reaching these breakthroughs requires acknowledging that much still remains to be explored at the very foundations of Neuromechanobiology. In this context, and as discussed throughout this review, we are still far from fully understanding how mechanosensing and mechanotransduction processes modulate or fundamentally contribute to neuronal development, neuronal activity, and their pathological alterations across multiple biological dimensions. The review highlights several open questions and outlines potential avenues to address these emerging challenges, including loss of function experiments, transcriptomic analysis or strategies based on the controlled and context-specific modulation of tissue and matrix stiffness during developmental and pathological processes of neurons and glial cells (e.g., [292,354,374]). In addition, to advance our understanding of these processes, it is essential to clearly identify the limitations of current 2D and 3D experimental models, particularly in light of the fact that cellular mechanobiological responses in 2D systems often differ substantially from those observed in 3D environments. Recognizing these differences is crucial to deriving a more comprehensive and integrated view of Neuromechanobiology (e.g., [228,375,376,377,378]). In this regard, the synergy between different scientific disciplines—including neuroscience, mechanobiology, physics, bioengineering, and computational modeling—has become, and will continue to be, a fundamental framework for disentangling these complex mechanisms and for establishing their biological relevance across development, function, and disease.

## Data Availability

No new data were created or analyzed in this study. Data sharing is not applicable to this article.

## Abbreviations

The following abbreviations are used in this review:

ECM	Extracellular matrix
AD	Alzheimer’s disease
MS	Multiple sclerosis
GBM	Glioblastoma multiforme
FAK	Focal adhesion kinase
YAP	Yes-associated protein
TAZ	Transcriptional coactivator with PDZ-binding motif
CSF	Cerebrospinal fluid
MSICs	Mechanosensitive ion channels
ENaC	Epithelial sodium channels
DEG	Degenerins
TRP	Transient receptor potential
K2P	Two-pore domain potassium channel
MDFIC	MyoD family inhibitor protein
DRG	Dorsal root ganglia
KCNK2	Potassium channel subfamily K member 2
KCNK10	Potassium channel subfamily K member 10
TRAAK	TWIK-related arachidonic acid-stimulated K^+^ channel
KCNK4	Potassium channel subfamily K member 4
TRPA	Transient receptor potential Ankyrin
TRPC	Transient receptor potential Canonical
TRPM	Transient receptor potential Melastatin
TRPML	Transient receptor potential Mucolipin
TRPN	Transient receptor potential NO-mechano-potential, NOMP
TRPP	Transient receptor potential Polycystin
TRPV	Transient receptor potential Vanilloid
TMEM150C	Transmembrane protein 150C
TMEM	Transmembrane protein
GPCRs	G-protein-coupled receptors
LPHN2	Latrophilin-2
AMPA	A -amino-3-hydroxy-5-methyl-4-isoxazolepropionic acid
FTD	Frontotemporal dementia
MST1/2	Mammalian Sterile 20-like protein kinase 1/2
SAV1	Salvador homolog 1
LATS1/2	Large Tumor Suppressor 1/2
AMOT	Angiomotin
SHH	Sonic hedgehog
NSCs	Neural stem cells
MAC	Methacrylamide chitosan
TUJ1	Type III β-tubulin isoform
NPC	Neural progenitor cell
aRG	Apical radial glia
IPs	Intermediate progenitors
oRG	Outer radial glia
CCE	Constrained cortical expansion
TIG	Tension-induced growth
MRE	Magnetic resonance elastography
GE	Ganglionic eminence
MEA	Microelectrode array
AFM	Atomic force microscopy
EEG	Electroencephalogram
TBI	Traumatic brain injury
